# Testing the Effectiveness of Environmental Variables to Explain European Terrestrial Vertebrate Species Richness across Biogeographical Scales

**DOI:** 10.1371/journal.pone.0131924

**Published:** 2015-07-10

**Authors:** Maud Mouchet, Christian Levers, Laure Zupan, Tobias Kuemmerle, Christoph Plutzar, Karlheinz Erb, Sandra Lavorel, Wilfried Thuiller, Helmut Haberl

**Affiliations:** 1 Univ. Grenoble Alpes, Laboratoire d’Ecologie Alpine (LECA), F-38000, Grenoble, France; 2 CNRS, Laboratoire d’Ecologie Alpine (LECA), F-38000, Grenoble, France; 3 Geography Department, Humboldt-Universität zu Berlin, Unter den Linden 6, 10099, Berlin, Germany; 4 Institute of Social Ecology Vienna, Alpen-Adria Universität Klagenfurt, Wien, Graz, Schottenfeldgasse 29, 1070, Wien, Austria; 5 Integrative Research Institute on Transformations of Human-Environment Systems (IRI THESys), Humboldt-Universität zu Berlin, Unter den Linden 6, 10099, Berlin, Germany; University of Brasilia, BRAZIL

## Abstract

We compared the effectiveness of environmental variables, and in particular of land-use indicators, to explain species richness patterns across taxonomic groups and biogeographical scales (i.e. overall pan-Europe and ecoregions within pan-Europe). Using boosted regression trees that handle non-linear relationships, we compared the relative influence (as a measure of effectiveness) of environmental variables related to climate, landscape (or habitat heterogeneity), land-use intensity or energy availability to explain European vertebrate species richness (birds, amphibians, and mammals) at the continental and ecoregion scales. We found that dominant land cover and actual evapotranspiration that relate to energy availability were the main correlates of vertebrate species richness over Europe. At the ecoregion scale, we identified four distinct groups of ecoregions where species richness was essentially associated to (i) seasonality of temperature, (ii) actual evapotranspiration and/or mean annual temperature, (iii) seasonality of precipitation, actual evapotranspiration and land cover) and (iv) and an even combination of the environmental variables. This typology of ecoregions remained valid for total vertebrate richness and the three vertebrate groups taken separately. Despite the overwhelming influence of land cover and actual evapotranspiration to explain vertebrate species richness patterns at European scale, the ranking of the main correlates of species richness varied between regions. Interestingly, landscape and land-use indicators did not stand out at the continental scale but their influence greatly increased in southern ecoregions, revealing the long-lasting human footprint on land-use–land-cover changes. Our study provides one of the first multi-scale descriptions of the variability in the ranking of correlates across several taxa.

## Introduction

Explaining biodiversity patterns remains one of the most challenging issues in ecology and evolution (e.g. [1,2]). Climatic conditions, energy availability and habitat heterogeneity have been put forward to explain the observed spatial and temporal macro-scale patterns of biodiversity (e.g. [3, 4, 5, 6, 7, 8]). The growing demand for food, materials, shelter and other ecological services from human societies has triggered major changes in landscapes worldwide [9]. Human activities may affect ecosystems in various ways from changes in climatic conditions or human use of lands and are thus likely to interact with climate (e.g. GHG emissions, heat-island effects), habitat heterogeneity (physical modifications of lands, fragmentation of ecosystems), or alter the availability of trophic energy in ecosystems through land use and through the diversion of trophic energy by and for human use. Land-use change and intensification can also affect ecosystems indirectly by disrupting the natural disturbance regime (e.g. fire suppression, alteration of flow regime) that has shaped species diversity [10]. Moreover, land-use change legacy might have a strong influence on current patterns of species richness, particularly in regions with a long history of land management [11]. Yet, the potential human impact on species richness at broad geographical scales has largely focused on the consequences of human-induced climate change but rarely on human-induced land-use change (but see [12]). Indeed, the effects of human-driven changes in land cover are mainly considered as local or regional issues.

The global latitudinal patterns of species richness are often associated with macroclimatic patterns but it is less certain that climate is the only determinant of species richness when focusing on the continental or smaller biogeographic units (e.g. ecoregions). The few studies that compared the ranking of the determinants of biodiversity patterns demonstrated that their relative influence may not be extrapolated from one biogeographic region to another [13]. Thus, global determinants of species richness may not be relevant at smaller biogeographic scales [14] and it is necessary to test their effectiveness to explain biodiversity patterns across biogeographic regions and across taxa.

Our overarching goal is to consolidate our knowledge on the scale-dependency of the ranking of species richness correlates. To achieve this goal, we compared the relative influence (also called “relative importance”) of various environmental variables on the explained variance of patterns of species richness of three vertebrate taxa (mammals, breeding birds, amphibians) at two biogeographical scales (i.e. continent and ecoregion). Considering its strong climatic gradient, Europe is a good candidate to determine which variables among climate, energy availability, habitat heterogeneity and land-use indicators best correlate with patterns of species richness. The European continent is not only densely populated but has also a long history of human land use resulting in highly diversified landscapes. This diversity of European landscapes is appropriate to include anthropogenic pressures and to look for land-use legacy on species richness. Then, climate may be the main determinant of species richness in northern European countries dominated by boreal forests, but less important in determining species richness patterns in the urban-rural mosaic of the more densely populated parts of central Europe. We thus expect that patterns of species richness should be primarily correlated to climatic patterns at the continental scale but that, in contrast, variables depicting energy availability and habitat heterogeneity might prevail at the ecoregion scale. Additionally, we hypothesise that the mechanisms underpinning species richness patterns might vary between taxonomic groups. In particular, we expect that amphibians, being ectothermic species, should be first correlated to patterns of temperature regardless of scale, while patterns of mammals and breeding birds, being endothermic species, could be correlated to patterns of energy availability or habitat heterogeneity at the ecoregion scales.

Climatic conditions, energy availability, environmental heterogeneity and land-use-land-cover characteristics will be represented by a set of environmental variables (e.g. mean temperature, the amount of net primary productivity (NPP), topographic heterogeneity). The potential influence of human activities on species richness and the potential interactions of human activities with habitat will be accounted for using various landscape metrics. The diversion of ecosystem productivity for human use will be taken into account by incorporating the Human Appropriation of Net Primary Production, a measure of human impacts on the availability of NPP in ecosystems resulting from land use (HANPP, [15]).

## Materials and Methods

### Biodiversity distribution data

We used data on 275 mammals, 429 breeding birds and 102 amphibians that were compiled from Maiorano and colleagues ([16]) and cover the European sub-continent, including Turkey. For mammals and amphibians, the primary data were extent of occurrences (EOO) collected from the IUCN Global Mammal and Amphibian Assessments (http://www.iucnredlist.org/initiatives/mammals and http://www.iucnredlist.org/initiatives/amphibians, available on the IUCN website). For bird species, data on EOOs available from Hagemeijer and Blair ([17]) were combined with those available from the BWP*i*2.0.1 DVD-ROM (Birds of the Western Palearctic interactive 2006, version 2.0.1). For each taxon, EOOs were refined using species’ habitat preferences (based on both expert knowledge and literature). Habitat preferences of each species was expressed as a suitability score (0: unsuitable, 1: secondary, 2: primary habitat) assigned to each GlobCover land-use land-cover class. Following scores, EOOs were filtered out at a high resolution (i.e. 300m) to remove false presence data (no presence data added). Then, filtered EOOs were up-scaled to a lower resolution (i.e. 10’), more suitable to biogeographical studies. Indeed, the up-scaling step limits the fine-scale signal of land-cover on biodiversity data and enables further comparisons with outcomes from Maiorano et al. [16]. A species was considered present in a 10’ cell as long as it occurred at least in one of the overlapped 300m cells. The reliability of filtered EOOs of 37% of the amphibians, 71.4% of the birds and 33.8% of the mammals from the final species list, were also evaluated against field data. Refined EOOs evaluated for amphibians and mammals performed very well (see Maiorano et al. [16] for further details on the filtering and evaluation processes). All species’ distribution data followed a regular grid of 10’ resolution (WGS 84). Resulting maps were overlaid and summed to estimate species richness per pixel for the three groups of species individually and combined, to estimate the total vertebrate species richness.

### Environmental variables

Based on the extensive literature discussing the relevance of various variables to explain macro-scale species richness patterns (e.g. [5,13,18–20]), we selected a set of environmental variables related to climate, energy availability and habitat (or environmental) heterogeneity: annual mean temperature (Bio1), temperature seasonality (Bio4), annual precipitation (Bio12), and precipitation seasonality (Bio15), net primary productivity left after harvest (NPP_eco_ *sensu* [21]), actual evapotranspiration (AET) and terrain ruggedness (TRI). In particular, we included several landscape structure indices to account for habitat variability: land cover diversity (GLC_simp), patch size coefficient of variation (patchSize), aggregation index (Aggreg) and dominant land cover type (GLC_maj). This latter variable will help discriminate which type of land cover supports a given level of NPP_eco_ and AET. Finally, the human appropriation of net primary productivity (i.e. HANPP, [15]) was included to account for human impact on landscapes. The variables are listed and briefly described in Table 1.

**Table 1:** Environmental variables characterizing macro-scale hypotheses for biodiversity patterns.

	Code	Description	Unit	Relevance	Data source
Human Appropriation of Net Primary Production	HANPP	Mean land-use intensity for the year 2000 estimated at 10' from a 5' grid	tC/yr	HANPP integrates many sources of anthropogenic pressures (agricultural intensification, urbanisation, etc.) that affect the amount of trophic energy available for wild-living species	[15, 71]
Net Primary Production left after harvest	NPPeco	Mean NPP left for the year 2000 estimated at 10' from a 5' grid	tC/yr	Represents the amount of energy converted into vegetal organic matter and available for free living consumers to turn into biomass	[15]
Actual evapotranspiration	AET	Quantity of water removed from a surface due to the processes of evaporation and transpiration	mm/yr	AET is directly related to vegetation productivity and represents the balance between water and energy	ATEAM
Dominant land cover type	GLC_maj	Calculated as the dominant global land cover (GLC) category in each 10' pixel of the reference grid	-	Defines which type of habitat is supporting NPP	Global Land Cover 2009 map, ESA-JRC
Land cover diversity	GLC_simp	Calculated as the Simpson's diversity of all 1km² land cover pixels falling in each 10' pixel of the reference grid	-	Is the related to the variability (or heterogeneity) of habitats and thus the complexity of the landscape	Global Land Cover 2009 map, ESA-JRC
Patch size coefficient of variation	patchSize	Variability is estimated as a percentage of the mean size of patches (here patches are the 1km² land cover pixels of GLC) in a given landscape (i.e. 10' pixel of the reference grid)	-	Helps to compare the relative variability of land cover types among landscapes	Global Land Cover 2009 map, ESA-JRC
Aggregation index	Aggreg	Calculated as the mean of aggregation index value of all land cover types in a 10' pixel	-	Depicts the tendency of patch types to be spatially aggregated that is landscape texture	Global Land Cover 2009 map, ESA-JRC
Terrain ruggedness	TRI	Topographic heterogeneity based on amount of elevation difference between adjacent cells	m	Is related to the variability of elevation in a given location (i.e. a 10’ pixel)	[72] using SRTM30 data
Annual mean temperature	Bio1	Annual mean temperature for the 1960–90 period	°C	Is related to the amount of solar energy available in an ecosystem that is assumed to influence evolutionary rates and the balance between thermoregulation and growth or reproduction	WorldClim Global Climate Data
Seasonality of temperature	Bio4	Based on the standard deviation of temperature for the 1960–90 period	°C	Define the climatic stability of a location	WorldClim Global Climate Data
Annual precipitation	Bio12	Annual trends of precipitation for the 1960–90 period	mm	Relates to the amount of energy available	WorldClim Global Climate Data
Seasonality of precipitation	Bio15	Coefficient of variation of annual precipitations for the 1960–90 period	-	Defines the climatic stability of a location	WorldClim Global Climate Data

We analysed whether our variables were strongly correlated to ensure that collinearity was minimized at the continental and ecoregion scale. The mean Spearman’s correlation coefficient between pairs of variables was low at the continental scale and at the ecoregion scale (i.e. mean absolute correlation coefficient of 0.23, with a standard deviation of 0.20), with the strongest correlation coefficients being between actual evapotranspiration (AET) and annual precipitation (Bio12), HANPP and annual mean temperature (Bio1), or between patch size variability (patchSize) and land cover diversity (GLC_simp).

### Geographical extent and reference grid

Following Mücher and collaborators ([22]), we divided the pan-European continent (including Turkey) into 15 ecoregions (S1 Fig), representing a wide variety of environmental conditions. These ecoregions were derived from the Environmental Stratification of Europe, based on climate and geomorphology [23].

Over the area studied, species richness and environmental variables were summarized for a 10' resolution reference grid mapped in the 1984 version of the World Geodetic System (WGS 84), using ArcGIS 10.0 and R.3.0.1. ([24]). For variables mapped at a finer scale than 10’, pixel values were aggregated at the 10’ resolution using the mean value in the case of HANPP, NPP_eco_ and Aggreg, or the dominant value in the case of GLC_maj (see Table 1 for further details).

### Analysing species richness patterns

We used boosted regression trees (BRTs) [25] to explain the variability of species richness across Europe and to quantify the relative influence (“relative importance” in [26]) of the predictors, a.k.a the environmental variables. BRTs belong to the family of non-parametric, machine learning models, which make no assumptions as to the distribution of target or explanatory variables. Machine learning methods have several advantages over statistical models such as their robustness in the face of missing and collinear data, their ability to handle non-linear relationships and to address variable interactions [26–28]. BRTs are built upon regression trees, which explain the variance of a target variable by fitting simple models on partitions of the entire data space. These recursive partitions are derived by splitting up the data space into groups that are as homogeneous as possible in terms of response and that minimise prediction errors. Afterwards, BRTs combine many simple decision trees in an ensemble (i.e. boosting) by adding trees in a forward and stage-wise fashion to minimise the loss function of the model [26]. We built BRT models with the whole set of explanatory variables to explain patterns of total species richness and individual taxa species richness in (1) a global, pan-European, approach and (2) stratified by ecoregions. The calibration of BRTs requires four parameters to be specified. First, the number of trees (nt), which defines the maximum amount of single decision trees on which the BRT model is built. Second, the tree complexity (tc), which defines the model complexity in terms of allowed interactions between predictors. Third, the learning rate (lr), which is a shrinkage parameter determining the contribution of each single decision tree within the entire BRT model. And fourth, the bag fraction, which defines the percentage of input data that is withheld while fitting the model to be used for testing [26–27]. After testing for parameter sensitivity, we set tree complexity to 2, learning rate to 0.01, bag fraction to 0.5 and used a Gaussian error distribution. The final number of trees kept in each BRT model was determined using a stepwise procedure as implemented in the *gbm*.*step* routine provided by the *dismo* package [29] in R [24]. The performance of the model was assessed using the percent of explained deviance [26]. The explained deviance of the BRT model was calculated as: 1 - (residual deviance/total deviance).

To interpret the results, we assessed the relative influence of each explanatory variable based on the number of times a given variable was selected for splitting a single tree, weighted by the squared improvements of error risk, and then averaged over all trees [30]. Each relative importance was finally standardized such that the sum adds up to 100. The contribution of variables to model fit was expressed as percentages and ranked to identify the most influential variables, i.e. the predictor(s) explaining the highest amount of species richness variability.

Subsequently, we generated partial dependency plots to interrogate the relationship between species richness and each explanatory variable [31–32]. The partial dependency plots show how a given explanatory variable influences the target variable (i.e. species richness) along its data range while controlling for the average effects of all other explanatory variables [33]. For better interpretability, we smoothed the response curves with a spline interpolation.

## Results

**Fig 1:**
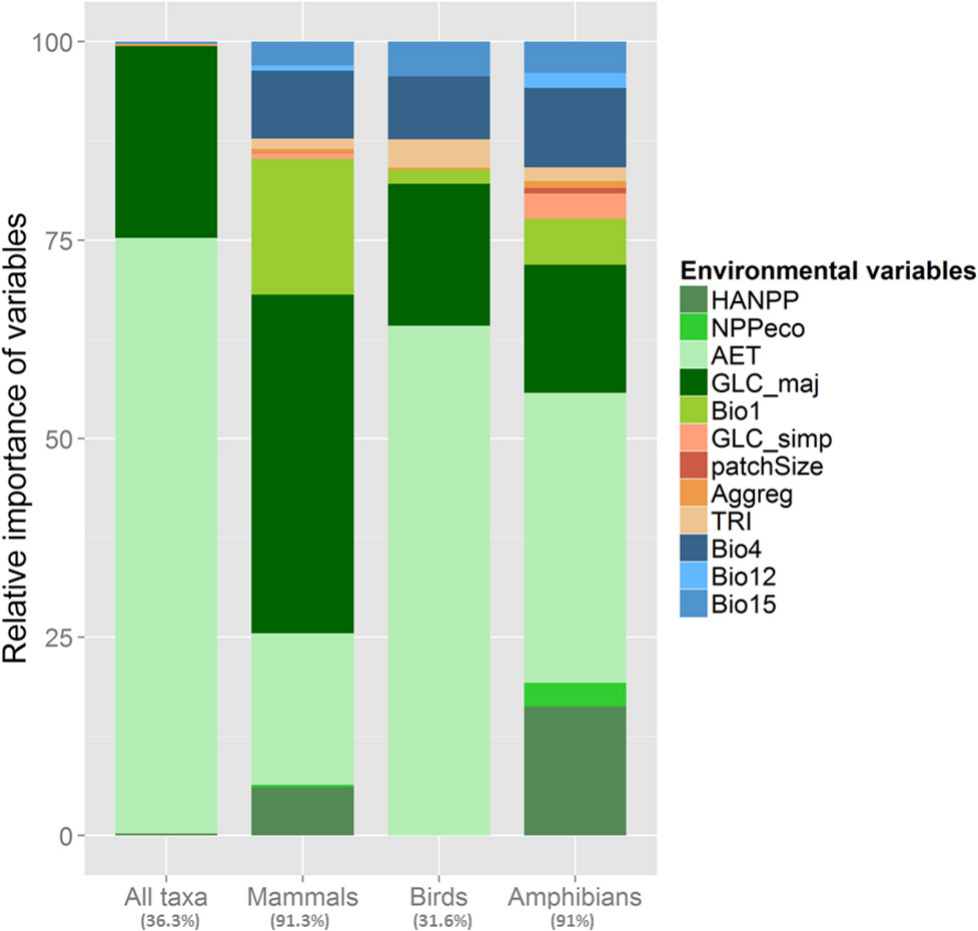
Relative contribution of environmental variables to explained variance of the Boosted Regression Trees models of patterns of species richness of each vertebrate group at the pan-European scale. Environmental variables are detailed in Table 1. Values in brackets are the performance of BRT model for each taxon and expressed as a percent of deviance explained (%).

Over pan-Europe, the variability of the richness of each taxon, and total species richness revealed to be best predicted by actual evapotranspiration (AET). However, the performance of the models describing patterns of total (36.3% of deviance explained by the model) and avian (31.6% of explained deviance) species richness, were significantly lower than the performance of the models relating to mammals (91.3% of explained deviance) and amphibians (91% of explained deviance) (Fig 1).

**Fig 2:**
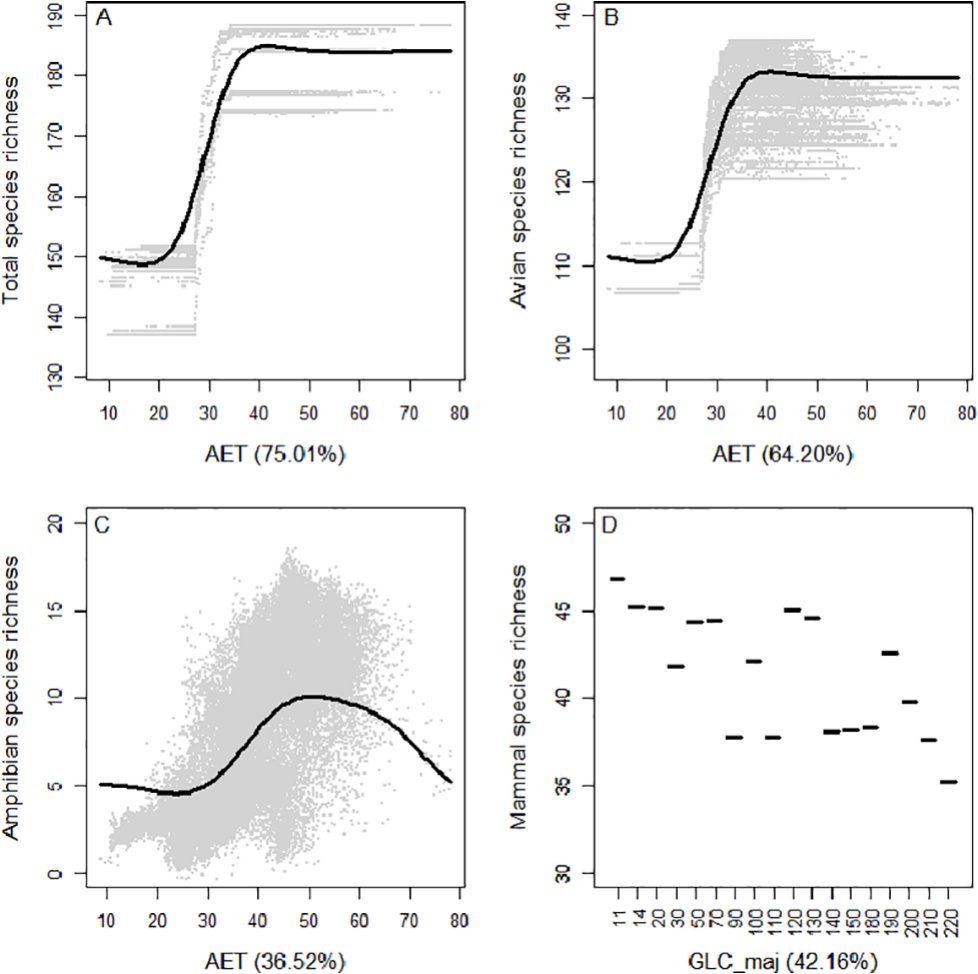
Partial dependency plot representing the relationships between the predicted species richness of all vertebrates (A), of birds (B), of amphibians (C) and of mammals (D) and the main contributors identified by BRT models, namely AET for A-C and GLC_maj for D, at the continental scale.

AET and the dominant type of land-use-land-cover, i.e. GLC_maj, explained the highest part of the spatial variability of the total species richness (i.e. 75% and 24.1% respectively, Fig 1). A sigmoid, yet positive, curve related AET to total species richness (see Fig 2A as an illustration). Total species richness strongly increased between 20 and 40 mm/yr of AET so that high levels of AET sustain higher species richness. The other environmental variables showed only a marginal influence on vertebrate species patterns (Fig 1).

Similarly, species richness patterns of amphibians and, especially, birds were better explained by AET (explaining 36.5% and 64.2% of the variability in amphibian and avian species richness patterns) and, to a lesser extent, GLC_maj (16.1% and 17.9% respectively). In the particular case of amphibians, the amount of energy appropriated by humans (HANPP) was also an important predictor of species richness (16.2%). Partial dependency plots showing avian species richness and AET revealed a sigmoid curve similar to the relationship between total species richness and AET (Fig 2A and 2B). Comparatively, the relationship between amphibian species richness and AET formed a hump-shaped curve with a maximum of predicted species richness at approximately 50 mm/yr of AET. Higher AET values relate to higher amphibian species richness, however, beyond approximately 60 mm/yr of AET predicted species richness abruptly decreases (Fig 2C).

Partial dependency plots showing the relationship between mammal richness and the main predictor, i.e. GLC_maj, showed that the number of mammal species was higher in pixels dominated by croplands (classes 11, 14 and 20), closed forests (classes 50 and 70), mosaic of grasslands, forests and shrublands (class 120) and closed to open shrublands (class 130) (see Fig 2D). Mammal species richness was lower in open habitats (classes 90, 110, 140), sparse (class 150) or regularly flooded (class 180) vegetation, water bodies (class 210) and snow (220) (see S1 Table for the description of classes).

**Fig 3:**
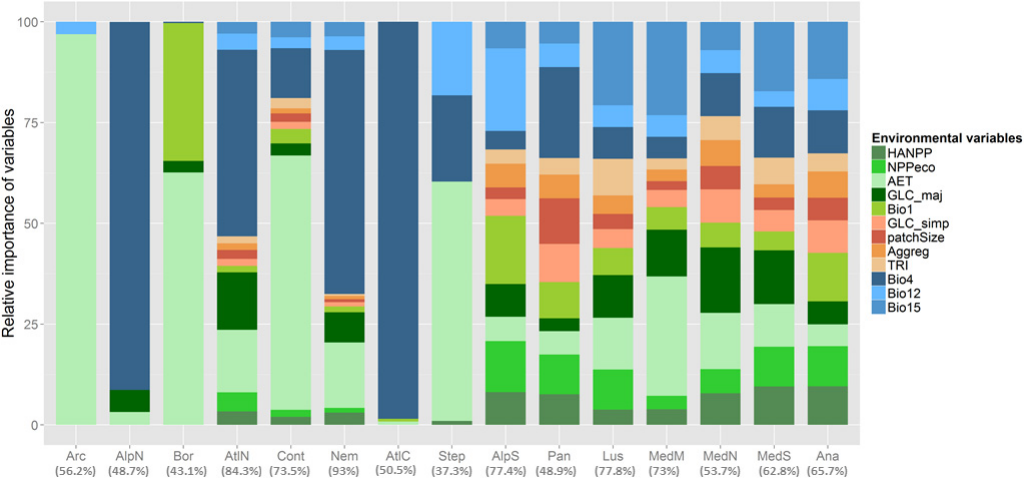
Relative influence of environmental variables on explained variance of patterns of terrestrial vertebrate species richness at the pan-European scale and by ecoregions modelled using Boosted Regression Trees. “Arc”: Arctic; “AlpN”: Northern Alpine; “Bor”: Boreal; “AtlN”: Northern Atlantic; “Cont”: Continental; “Nem”: Nemoral; “AtlC”: Central Atlantic; “Step”: Steppic; “AlpS”: Southern Alpine; “Pan”: Pannonian; “Lus”: Lusitanian; “MedM”: Mediterranean Mountains; “MedN”: Northern Mediterranean; “MedS”: Southern Mediterranean; “Ana”: Anatolian. Environmental variables are detailed in Table 1. Values in brackets are the performance of BRT model for each taxon and expressed as a percent of deviance explained (%).

**Fig 4:**
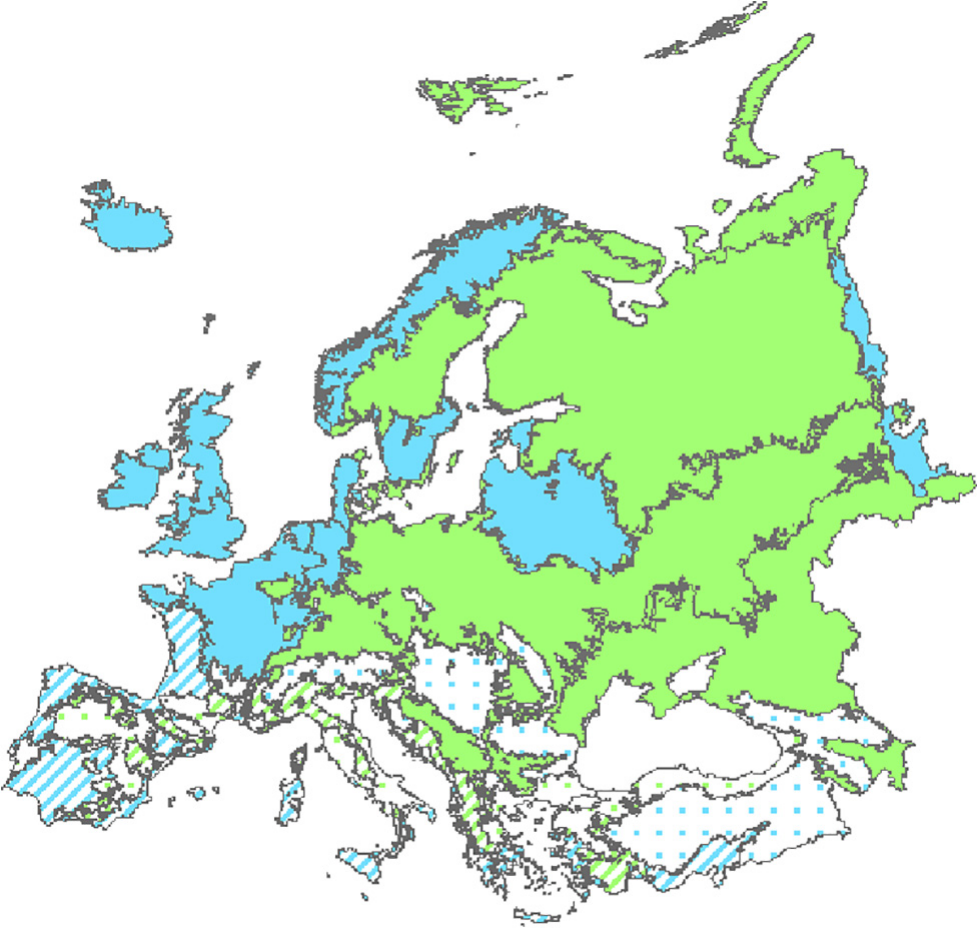
Clustering of ecoregions according to the best predictors of species richness patterns. The ecoregions are coloured according to the best explanatory environmental indicator: ecoregions where species richness patterns are best explained by AET are in green, by Bio4 in blue while green dots and dash blue lines represent ecoregions where best predictor is related to energy availability and climate respectively, but other predictors from the other hypotheses are almost as good predictors.

At the ecoregion level, BRT models yielded a high explanatory power as well (on average 63.1% with a standard deviation of 16.4%) but the relative contribution of the predictors strongly varied across ecoregions (Fig 3). AET was by far the best predictor of total species richness in Arctic, Boreal, Continental and Steppic ecoregions. Similarly, total species richness patterns in Northern Alpine, Nemoral, Central and Northern Atlantic ecoregions were strongly related to temperature seasonality patterns (Bio4, Fig 3). The amount of the deviance of species richness in the other ecoregions was more evenly distributed among environmental variables. Based on these results, we clustered ecoregions into four groups: (i) Arctic, Boreal, Continental, and Steppic ecoregions where AET explained 59.4% to 96.9% of species richness variability; (ii) Northern Alpine, Northern Atlantic, Nemoral, and Central Atlantic ecoregions where Bio4 explained 46.3% to 98.5% of the variability of total species richness; (iii) Southern Alpine, Pannonian, Anatolian, Lusitanian, and Southern Mediterranean regions which have in common that the most contributing predictor is related to climate (i.e. Bio4, Bio12 or Bio15) but had a more even contribution of all the other predictors; and (iv) Mediterranean Mountains and Northern Mediterranean ecoregions that have in common that variables related to energy availability (AET), land cover (GLC_maj) and seasonality (Bio15 and Bio4 respectively) are among the best predictors of species richness (Fig 4). Except for the first group, the ranking of predictors of total species richness by ecoregions significantly differed from the ranking of predictors at the continental scale. The results of the analyses performed by ecoregion and taxon, were comparable to the trends observed for total species richness. In order to remain concise, these results will not be described.

**Fig 5:**
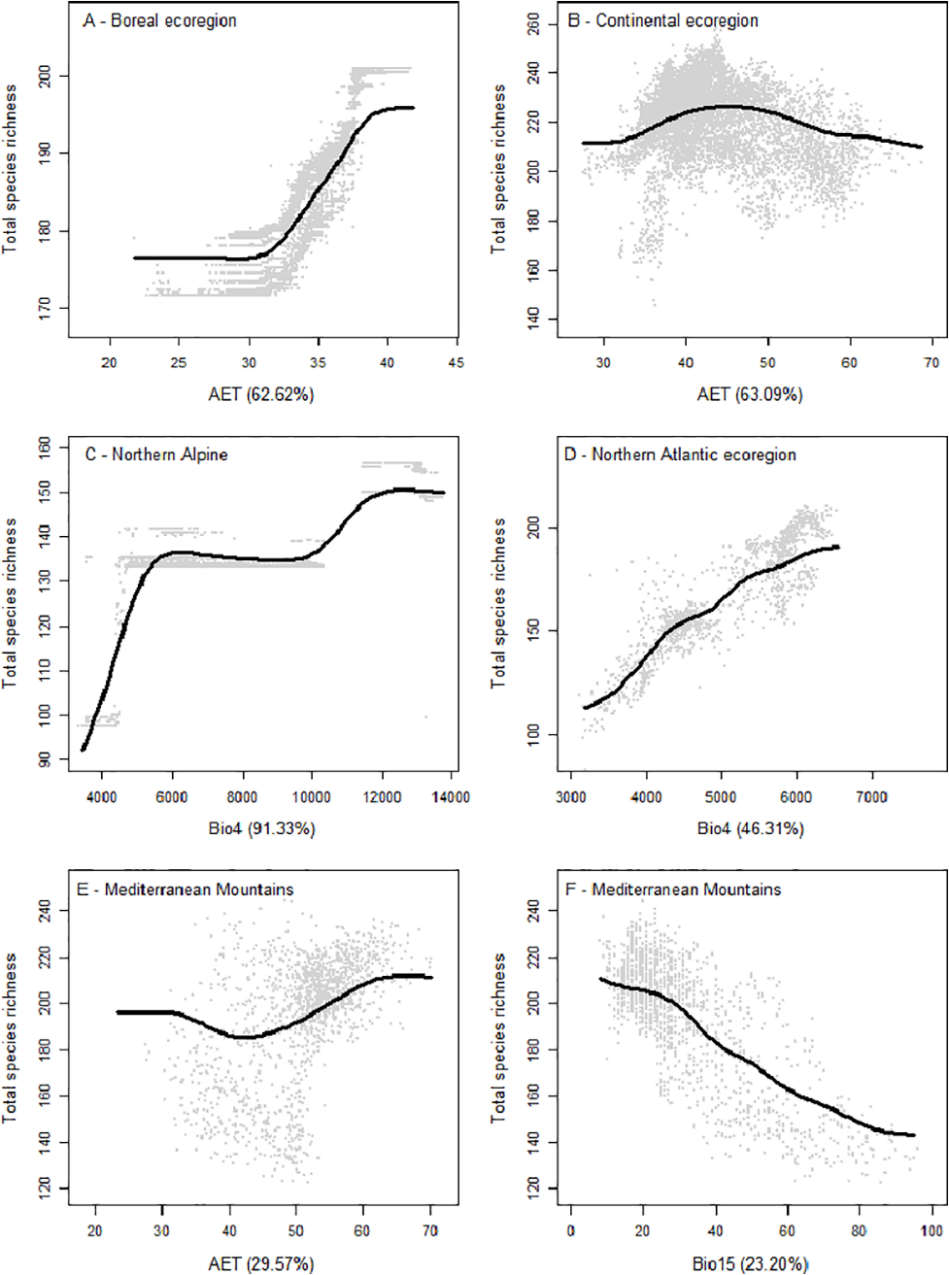
Examples of partial dependency plot representing the relationships between the predicted species richness of all vertebrates in Boreal (A), Continental (B), Northern Alpine (C), Northern Atlantic ecoregions (D) and Mediterranean mountains (E and F) and the main contributors identified by BRT models, namely AET for A, B and E, Bio4 for C-D and Bio15 for E.

Finally, our analyses showed that the shape of the relationships between species richness and predictors depended on the ecoregion and the variable considered. Partial dependency plots revealed a sigmoid relationship with a sharp increase of predicted species richness at an AET of 30 to 40 mm/yr approximately (illustrated in Fig 5A) for all ecoregions but the Continental and Mediterranean Mountains, which showed a decelerating or accelerating unimodal trend, respectively (Fig 5B and 5E). In Northern Alpine, Northern Atlantic, Nemoral and Central Atlantic regions, total species richness increased with temperature seasonality (Bio4) (Fig 5C and 5D). In the case of Lusitanian, Mediterranean mountains, and Southern Mountains, vertebrate richness showed a similar decreasing trend with precipitation seasonality (Bio15) (Fig 5F). Finally, in the case of Southern Alpine, Pannonian, Northern Mediterranean, and Anatolian ecoregions, the profiles of relative contributions of the different environmental variables were too diversified to find similarities among regions.

## Discussion

In the growing literature addressing biogeographical patterns of biodiversity, the strength of our study lies in the cross-analysis of the scale-dependency of species-richness correlates for three taxa while explicitly accounting for human appropriation of natural resources. We compared the ranking of environmental variables depicting the influence of climate, energy availability, habitat heterogeneity and land-use characteristics on patterns of species richness of terrestrial vertebrates across spatial scales (i.e. continent and ecoregions) and across taxa. Our finding that AET (an indicator of productive energy) was recurrently the best or one of the best predictors is in line with previous findings pinpointing that the “species-energy relationship” hypothesis dominated continental patterns of species richness at broad scales [34–37]. The energy hypothesis states that a positive species-energy relationship is frequently explained by the ability to support larger population sizes [38] through two alternative energy pathways, i.e. ambient (thermal) and productive (chemical) (see [39]). For instance, an increase in productive energy may result in more available resources (either in the diversity of resources or their amount), potentially more rare resources and, thus, facilitate co-existence of a larger number of specialist species (already proposed by [38]), a lower extinction risk, and longer food chains [18]. A recent meta-analysis [6] provides strong support for positive species-productivity relationships across diverse animal taxa. The overall pattern of species richness was also correlated to the dominant type of habitat (GLC_maj), mean annual temperature (Bio1, a climatic variable related to ambient energy) in the case of mammals, and HANPP in the case of amphibians. In contrast, the ranking of the predictors explaining variations in species richness at smaller scales differs according to the ecoregion considered supporting either the “species-energy relationship” (frequently represented by AET but also NPP) or the “climatic suitability” (commonly represented by temperature, precipitation and seasonality variables) hypotheses. The “climate suitability” hypothesis postulates that climate may drive biodiversity patterns through solar radiation that defines macroclimatic conditions of temperature and rainfall. Climate is also involved in the phylogenetic history of a biogeographic region [7] and is notably influencing species speciation, extinction, and dispersal [40–41] by increasing carrying capacities or because of species’ particular physiological requirements [42].

Our results support the statement by Cusens and colleagues ([6]) that the relationship between species richness and productivity is positive rather than “hump-shaped”. Indeed, the shape and the slope of the relationship between species richness and the best predictor differ between ecoregions. For instance, total species richness usually increases with AET but is clearly hump-shaped in the Continental region. Likewise, total species richness increases with mean annual temperature in most ecoregions but tends to decrease in Pannonian and Lusitanian regions or reveals a hump-shaped (or unimodal) form in Southern Alpine and Anatolian regions. Such hump-shaped curves are often attributed to the “intermediate disturbance hypothesis” which states that species richness is maximized by intermediate frequency and magnitude of disturbances [43] but in our case they indicate a nonlinear response of species richness to evapotranspiration and/or temperature. A strong competition at higher productivity levels can explain such decelerating curves, too. It is generally considered that biotic interactions act at a small scale and cannot be detected at the macro-scale but several works suggest the opposite [44–46].

Compared to Northern ecoregions clearly influenced by variables related to the “species-energy relationship” (i.e. AET) or the “climate suitability” (i.e. Bio4 and Bio1) hypotheses, the ranking of total species richness determinants is much more variable among southern ecoregions of Europe. Precipitations (i.e. Bio12 and Bio15) replace temperature seasonality (Bio4) as first correlates of species richness in Southern Alpine, Lusitanian, Southern Mediterranean and Anatolian ecoregions and are also influential in Mediterranean Mountains. More importantly, HANPP, NPP not harvested, the dominant land cover, and variables related to the habitat heterogeneity (i.e. GLC_simp and patchSize) have higher contributions in the Anatolian and Mediterranean ecoregions, than in the other parts of Europe. The “habitat heterogeneity” hypothesis assumes that complex habitats are susceptible to provide more diverse niches and ways of consuming resources resulting in a higher number of co-existing species [47–49]. Indeed, these ecoregions represent a wide range of habitats from mountainous landscapes in the Southern Alpine to steppe-like relief of the Pannonian ecoregion, and climates from the humid Mediterranean-like climate of the Lusitanian ecoregion to the dry Continental climate of the Pannonian ecoregion. However, habitat heterogeneity resulting from topographic variability may influence the distribution and accessibility of resources. As a consequence, species richness may increase with heterogeneity but then decrease as heterogeneity is too strong and disrupts accessibility to resources. The increasing contribution of HANPP or the “habitat heterogeneity” variables may originate from several phenomena. In mountainous ecoregions (Southern Alpine and Mediterranean mountains for instance), the amount of productive energy available for species, NPP_eco_, may be heterogeneously distributed due to elevation and topographic variability. The prevalence of habitat heterogeneity variables in the Mediterranean ecoregions, where energy availability may not be a limiting factor, could result from the complex mosaic of land uses and natural habitats shaped by the long-lasting impact of human activities [50]. Likewise the long history of diversified agricultural practices in Pannonian, Northern and Southern Mediterranean or Anatolian ecoregions, may explain the significant contribution of HANPP, NPP_eco_ and GLC_simp (i.e. variability of habitats) to spatial species richness patterns. The work by Falcucci and colleagues ([51]), showing that agricultural intensification resulted in a strong decline in farmland biodiversity and that urban footprint increased considerably in densely populated Italian regions, supports such results.

Among the three vertebrate taxa investigated, amphibians are most sensitive to their habitat characteristics and the subsequent loss of potential preys. The significant contribution of HANPP, compared to the other habitat indicators, suggests that patterns of amphibians are more sensitive to changes in primary production than habitat fragmentation at broader scales. However, the relationship between amphibians and HANPP is clearly positive at the continental scale, in the Northern and Southern Alpine, Pannonian and Southern Mediterranean ecoregions (S2 Fig). This strong relationship might result from the combination of productivity and water availability in many lowlands, which supports human activities as well as many species, including amphibians. Species richness patterns of birds were best explained by the productive energy and, in particular, AET which is an indicator of water-energy balance (i.e. the balance between solar energy and water availability necessary for plant productivity, [34]). The strong spatial overlap between birds and water-energy balance predominates in the Arctic and Steppic regions (S3 Fig), characterized by sparse tree-less vegetation and harsh climatic conditions (hard frost in Arctic, alternation of frost and drought in Steppic). The model performance was lower in the case of birds, the most species-rich taxon of the three, than for other taxa or total species richness, which may result from a lower spatial variability of avian richness.

Even if all taxa and total species richness are primarily correlated to the “species-energy relationship” variables, the variables ranking is not entirely similar. Our results show that birds are mainly affected by productive energy (AET). So are amphibians but they are also responding to HANPP and the dominant land cover. On the other hand, spatial patterns of mammals are first correlated to land cover. Clearly, the three taxa respond the macro-scale hypothesis in a different way and ecological conclusions on spatial patterns of species richness may not be generalized to individual taxon or to other spatial scales as argued by Belmaker & Jetz ([14]).

The “species-energy relationship”, “climate suitability” and “habitat heterogeneity” hypotheses are difficult to disentangle because they are not mutually exclusive. In that sense, our work highlights the synergy of macro-ecological mechanisms which have been little described in previous biogeographical studies (but see [20,52]). For instance, species’ physiological tolerance to climatic conditions increases with solar energy [53]. Temperature, a climatic parameter, estimates ambient (solar) energy that influences species range through physiological or metabolic constraints. Likewise, AET is suggested as a proxy of productive energy but relies on both solar (so ambient) energy and water-energy balance. This duality is particularly relevant for amphibians that are very sensitive to the combination of ambient energy (i.e. temperature) and moisture (related to precipitations) ([36,54]). Climatic conditions also influence vegetation patterns and, to a certain extent, the dominant type of land cover, which, in return, may have consequences on vertebrate patterns through energy availability and habitat characteristics [55–56]. For instance, the species-energy relationship is interrelated to climate to drive the geographical distribution of energy. The stability in climatic conditions partly determines primary producers and water-energy balance (commonly estimated by evapotranspiration), and through them, ecosystem productivity and animal species richness as well [34–35,57]. In return, habitat heterogeneity might play a role in the maintenance of primary producers and water-energy balance. All these mechanisms are related and may operate at different scales of observation [4,20,58].

Non-experimental approaches rarely establish unambiguously cause-and-effect relationships and we cannot exclude that our conclusions on the ecological processes driving species richness patterns are too simplistic. Besides the conclusions drawn here depend on how potential predictors (or correlates) of vertebrate species richness are imputed to one of these hypotheses. Annual mean temperature (Bio1) has been alternatively classified as a climatic factor or energy availability indicator. Annual precipitation (Bio12) could be considered as a factor for water-energy balance (energy availability). Precipitation seasonality (Bio15) may well be considered has a climatic factor or an environmental heterogeneity variable. The little contribution of spatial heterogeneity proxies may be explained by a scale discrepancy between our study and the relevant scale for habitat heterogeneity to shape species richness patterns. Another explanation to this lies in the high difficulty to delineate habitat heterogeneity that varies across scales: from the mosaic of small patches to topographic heterogeneity [49] but also from physical (e.g. topography, vegetation structure) to climatic variability (seasonality) that is usually associated to species-climate stability, another species richness theory. Another explanation is given by Fløjgaard et al. ([59]) who found that heterogeneity may principally influence endemic or widespread European mammal species but not the total species richness, the latter being mainly determined by macroclimatic conditions. In our case, the sites with the highest number of species locally co-occuring (i.e. within a 10’ pixel) identified by Maiorano and colleagues ([16]; see Fig 5 in their article) overlap well with mountainous landscapes (i.e. Mediterranean mountains, Lusitanian and Southern Alpine regions). The rapid changes in topography and land cover in such landscapes occur over very short distances fostering endemicity and speciation rate, often leading to a species richness peak at intermediate elevation ([60–61] and references therein).

It is worth noting that current biodiversity patterns are also influenced by past changes that may have occurred as long ago as the beginning of the past century [62–63]. This has been demonstrated for several taxa (e.g. plants, [64–65]; several trophic levels, [66]; birds, [67]). The accelerating rate of conversion of natural land cover to agricultural fields, urban areas or other types of exploitation, deeply altered habitat characteristics (e.g. energy availability, biotic corridors) and sustainably imprinted current and future biodiversity [63]. As for many species, productivity, resources availability and climatic conditions beneficial for human life, determine human use of land. Consequently, facing climate change and a growing human population, the response of biodiversity to changes may also vary, in space and time, according to future climate- and human-driven transitions of land use. While it is expected that the number of species will decrease at the global scale [68], species richness may increase in cooler regions experiencing warming, or arid regions experiencing more moisture availability. If determinants of biodiversity patterns are susceptible to change from one ecoregion to another and across taxa, a better understanding of the spatial repartition of forces driving species richness is required to draw relevant policies about biodiversity conservation. Preserving the water-energy balance at the continental scale may not be the most relevant strategy to protect mammals at the continental scale or limit the loss of overall vertebrates in the Northern Alpine ecoregion, where total species richness is first influenced by temperature seasonality. In other words, policies designed at a continental scale should probably be adapted at ecoregion scales and according to the taxon targeted. The relevance and operationalization of continental-wide biodiversity policy at smaller scale is crucial to halt biodiversity loss and should be a starting point for future research. In particular, in a context of growing urbanization, increasing knowledge on relationships between urban and non-urban biodiversity will certainly become a cornerstone to promote regional and continental biodiversity [69–70].

## Supporting Information

S1 Fig European ecoregions after Fig 1 in Mücher et al. (2009). Mücher CA, Hennekens SM, Bunce RGH, Schaminee JHJ, Schaepman ME (2009) Modelling the spatial distribution of Natura 2000 habitats across Europe. Landscape Urban Plan. 92(2): 148–159.
(TIF)

S2 Fig Partial dependency plots representing the relationships between the predicted species richness of amphibians and HANPP in four ecoregions. (TIF)

S3 Fig Partial dependency plots representing the relationships between the predicted species richness of birds and AET in two ecoregions. (TIF)

S1 Table Legend of land cover categories of GlobCover 2009. Further details are available in the updated Product Description and Validation Report of GlobCover 2009 (2011) at http://due.esrin.esa.int/globcover/.
(DOCX)
