# Supplementary figures and images for: Testing the Effectiveness of Environmental Variables to Explain European Terrestrial Vertebrate Species Richness across Biogeographical Scales
Source: PLoS One. 2015 Jul 10;10(7):e0131924. doi: 10.1371/journal.pone.0131924 (PMC4498906; doi:10.1371/journal.pone.0131924)

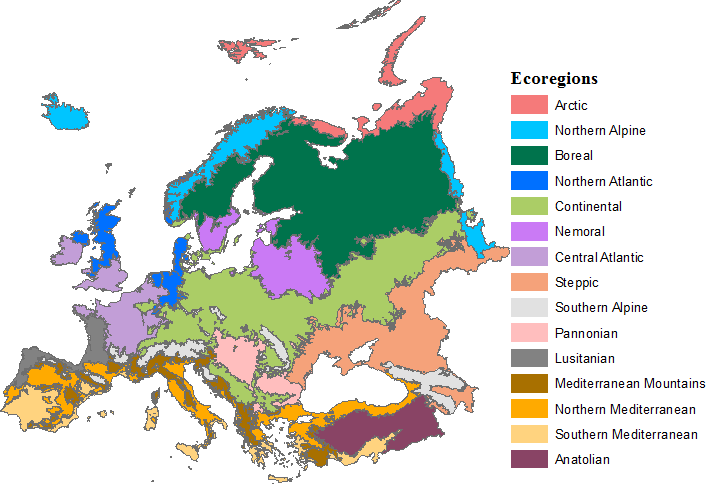

Supplement: S1 Fig — (2009). Mücher CA, Hennekens SM, Bunce RGH, Schaminee JHJ, Schaepman ME (2009) Modelling the spatial distribution of Natura 2000 habitats across Europe. Landscape Urban Plan. 92(2): 148–159. (TIF) [file pone.0131924.s001.tif]

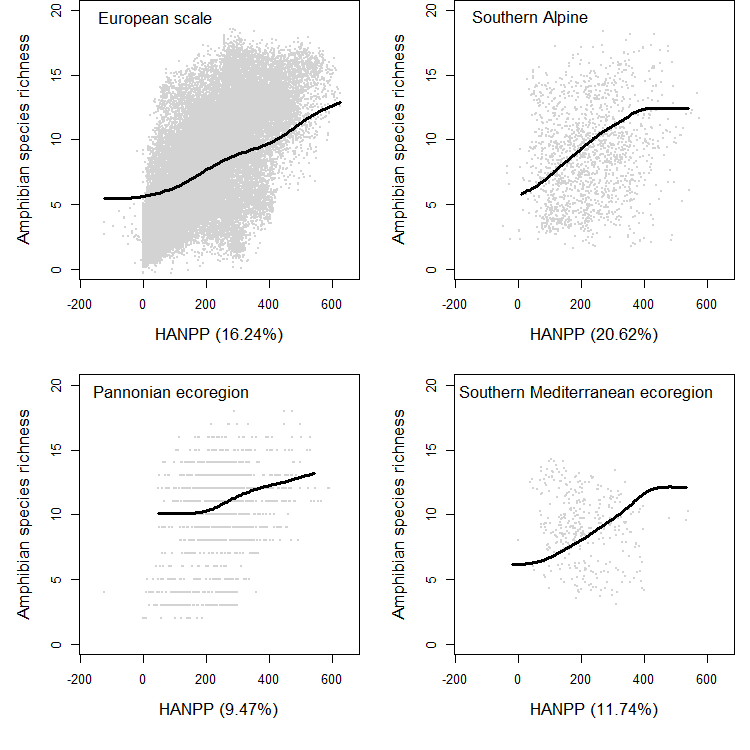

Supplement: S2 Fig — (TIF) [file pone.0131924.s002.tif]

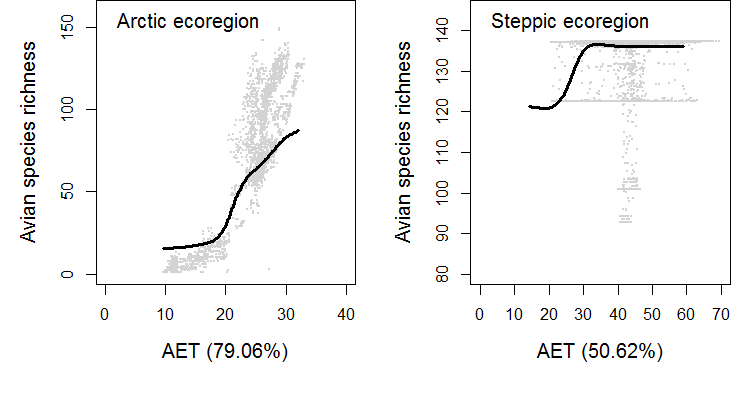

Supplement: S3 Fig — (TIF) [file pone.0131924.s003.tif]
