# Supplementary material for: Testing the Effectiveness of Environmental Variables to Explain European Terrestrial Vertebrate Species Richness across Biogeographical Scales
Source: PLoS One. 2015 Jul 10;10(7):e0131924. doi: 10.1371/journal.pone.0131924 (PMC4498906; doi:10.1371/journal.pone.0131924)
Supplement: S1 Table — Further details are available in the updated Product Description and Validation Report of GlobCover 2009 (2011) at http://due.esrin.esa.int/globcover/. (DOCX) [file pone.0131924.s004.docx]

**S1 Table. Legend of land cover categories of GlobCover 2009.**

Further details are available in the updated Product Description and Validation Report of GlobCover 2009 (2011) at http://due.esrin.esa.int/globcover/

| Code | Land cover type |
| --- | --- |
| 11 | Post-flooding or irrigated croplands |
| 14 | Rainfed croplands |
| 20 | Mosaic cropland (50-70%) / vegetation (grassland, shrubland, forest) (20-50%) |
| 30 | Mosaic vegetation (grassland, shrubland, forest) (50-70%) / cropland (20-50%) |
| 50 | Closed (>40%) broadleaved deciduous forest (>5m) |
| 70 | Closed (>40%) needleleaved evergreen forest (>5m) |
| 90 | Open (15-40%) needleleaved deciduous or evergreen forest (>5m) |
| 100 | Closed to open (>15%) mixed broadleaved and needleleaved forest (>5m) |
| 110 | Mosaic forest/shrubland (50-70%) / grassland (20-50%) |
| 120 | Mosaic grassland (50-70%) / forest / shrubland (20-50%) |
| 130 | Closed to open (>15%) shrubland (<5m) |
| 140 | Closed to open (>15%) grassland |
| 150 | Sparse (>15%) vegetation (woody vegetation, shrubs, grassland) |
| 180 | Closed to open (>15%) vegetation (grassland, shrubland; woody vegetation) on regularly flooded or waterlogged soil – fresh brackish or saline water |
| 190 | Artificial surfaces and associated areas (urban areas > 50%) |
| 200 | Bare areas |
| 210 | Water bodies |
| 220 | Permanent snow and ice |
